# Therapeutic Observations and Engagement Collaborative Project: Reducing the Number of Enhanced (1:1) Observations and Improving the Engagement on an Acute Inpatient Mental Health Ward

**DOI:** 10.1192/bjo.2026.11431

**Published:** 2026-06-30

**Authors:** Theodoros Mavrogiannidis, Tayo Lasisi, Isaac Obeng, Joao Manuel Sanches Rodrigues, Alessandro Malfatto

**Affiliations:** CNWL NHS Foundation Trust, London, United Kingdom

## Abstract

**Aims:**

Close Observations (1:1) are used as a measure of safety to closely monitor patients who are acutely unwell and with significant risks to their health, their safety, and the safety of others. However, the lack of substantial patient-staff engagement and continuous Multidisciplinary Team assessment can lead to an unnecessarily prolonged state of close observation. We aim to reduce the number of Close Observations on Pine Ward by 10% by the end of October 2025.

**Methods:**

This was a trust-wide project that lasted for one year, starting in November 2024. Our Multidisciplinary Team tried numerous change ideas using the Plan–Do–Study–Act framework. The change ideas that proved effective were the following: i) Increasing the number of structured therapeutic activities to subsequently improve patient experience, and reduce the number of 1:1 continuous observations. ii) Introduction of the Boredom Breaker Box to alleviate boredom in the patient experience. The box contains several activities ofinterest, i.e., comics, card games, and painting equipment.

**Results:**

Results regarding the data of enhanced observations were gathered through the Trust's tabbed journal. Questionnaires regarding patients' and staff experience were circulated quarterly throughout the year. The following results were produced: i) Number of unique patients onClose Observationsreduced from 10 to 6 (40%) per month by October 2025. ii) Overall, 49% improvement in patients' experience on the ward (increase from an average satisfaction of 3.14 to 4.68). iii) Overall, 20% increase in staff confidence to engage patients in 'boredom breaker' activities.

**Conclusion:**

The involvement of staff in introducing games and new activities on the ward, along with increased participation from both staff and patients, has helped alleviate boredom, improved patient productivity and engagement, and significantly reduced the number of 1:1 enhanced observations. Limitations in our project include: patients’ dynamics, increased patient flow, over-stimulation from high acuity on the ward, and the use of temporary staffing. Sustainability plans include: a poster with planned structured activities placed in different strategic areas on the ward, an allocated activity champion, a weekly patient care plan review to inform activity of interest, and daily check and handover of the boredom breaker box.

